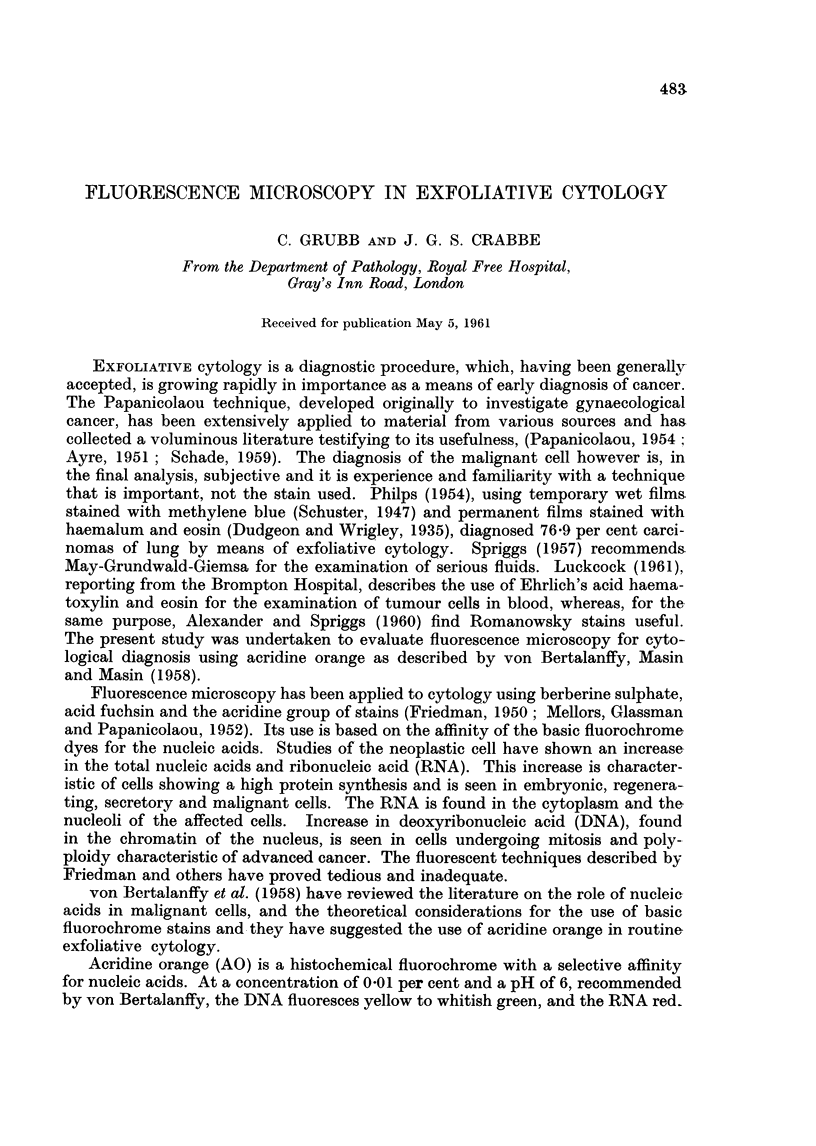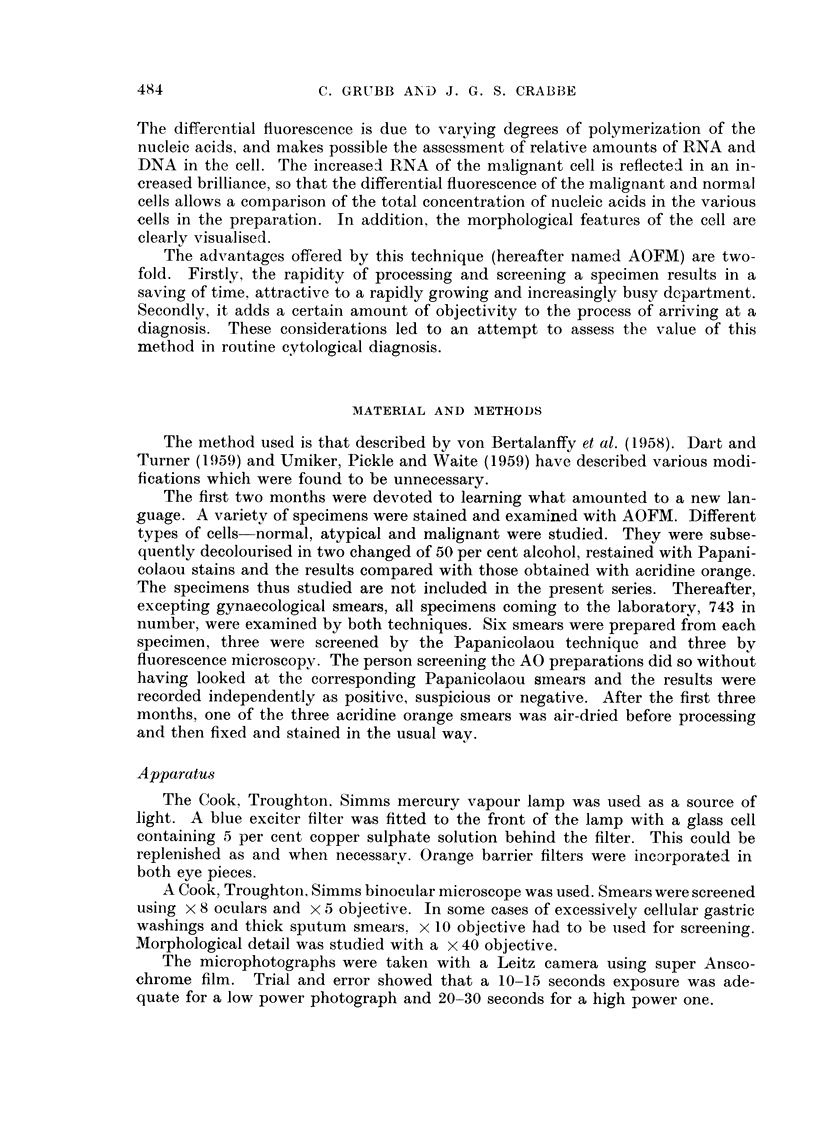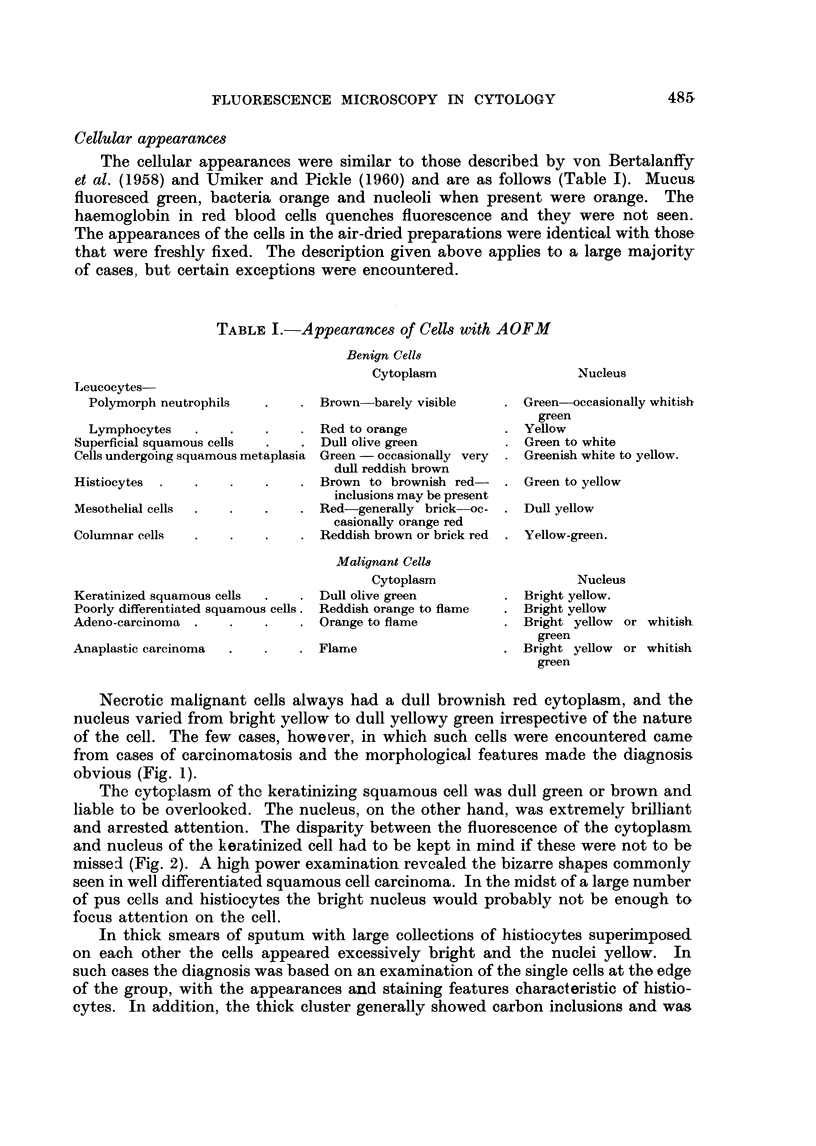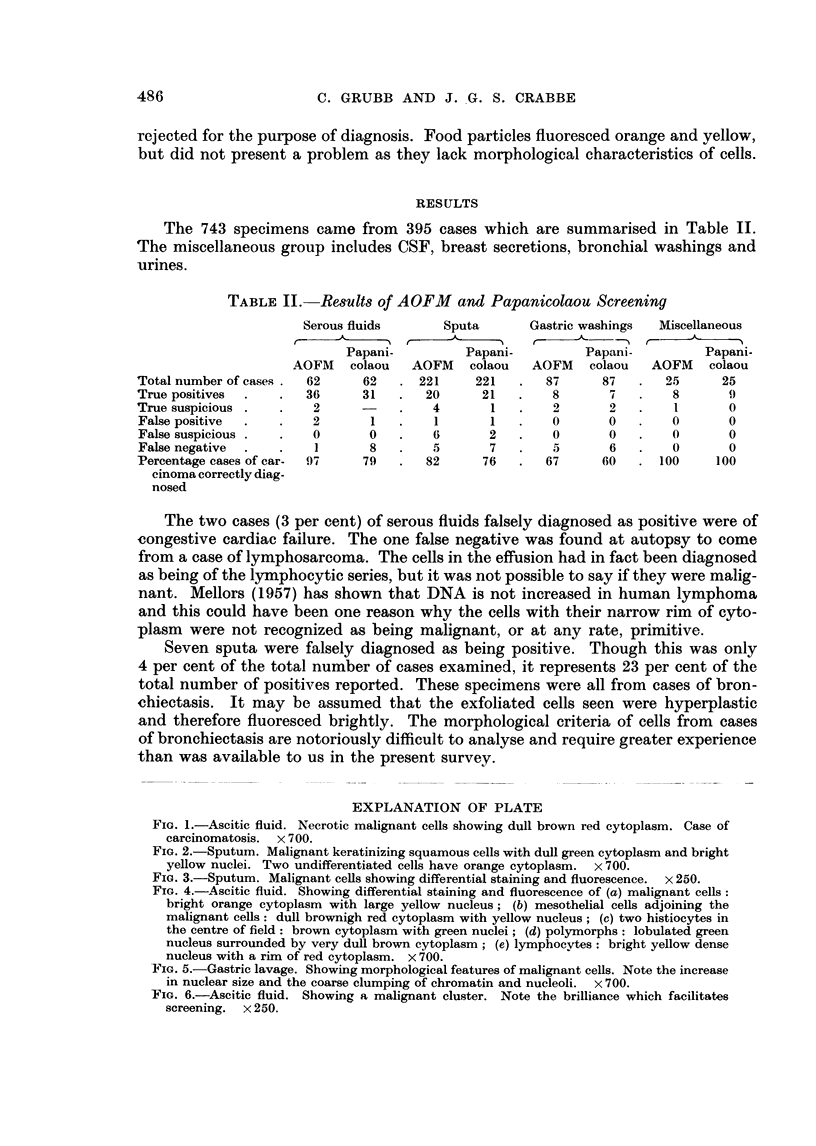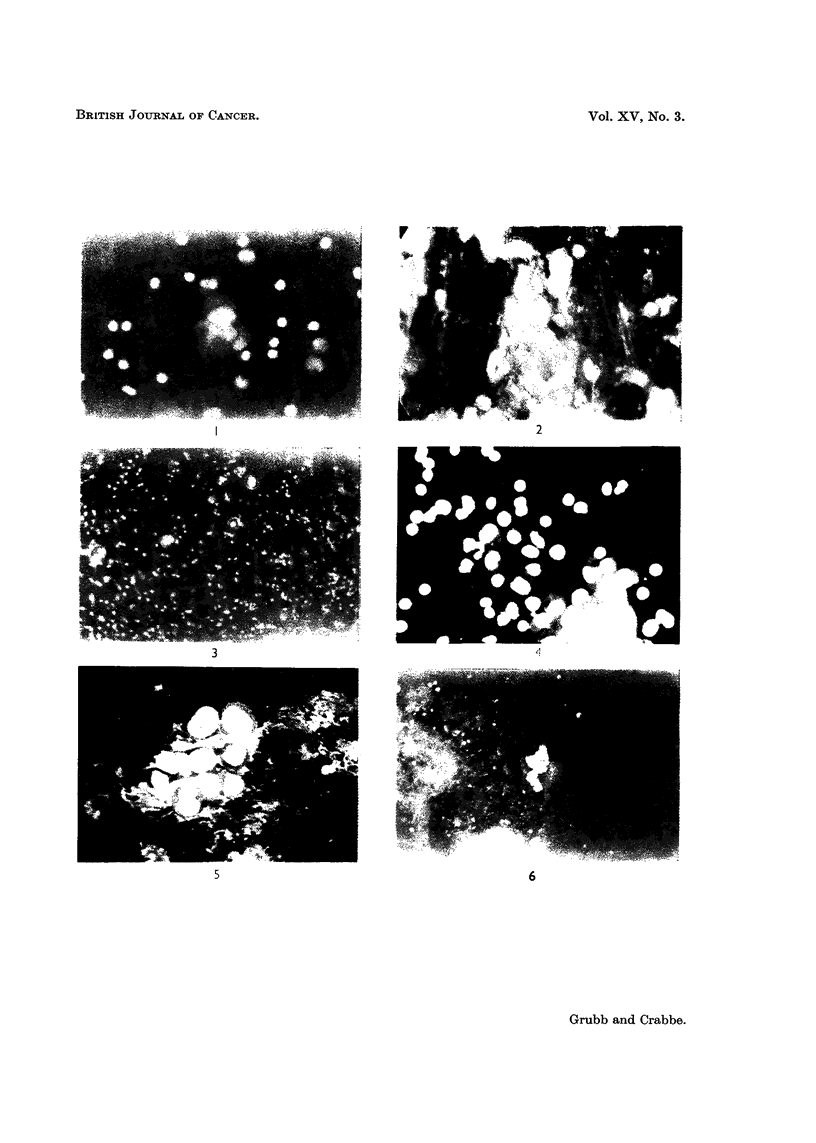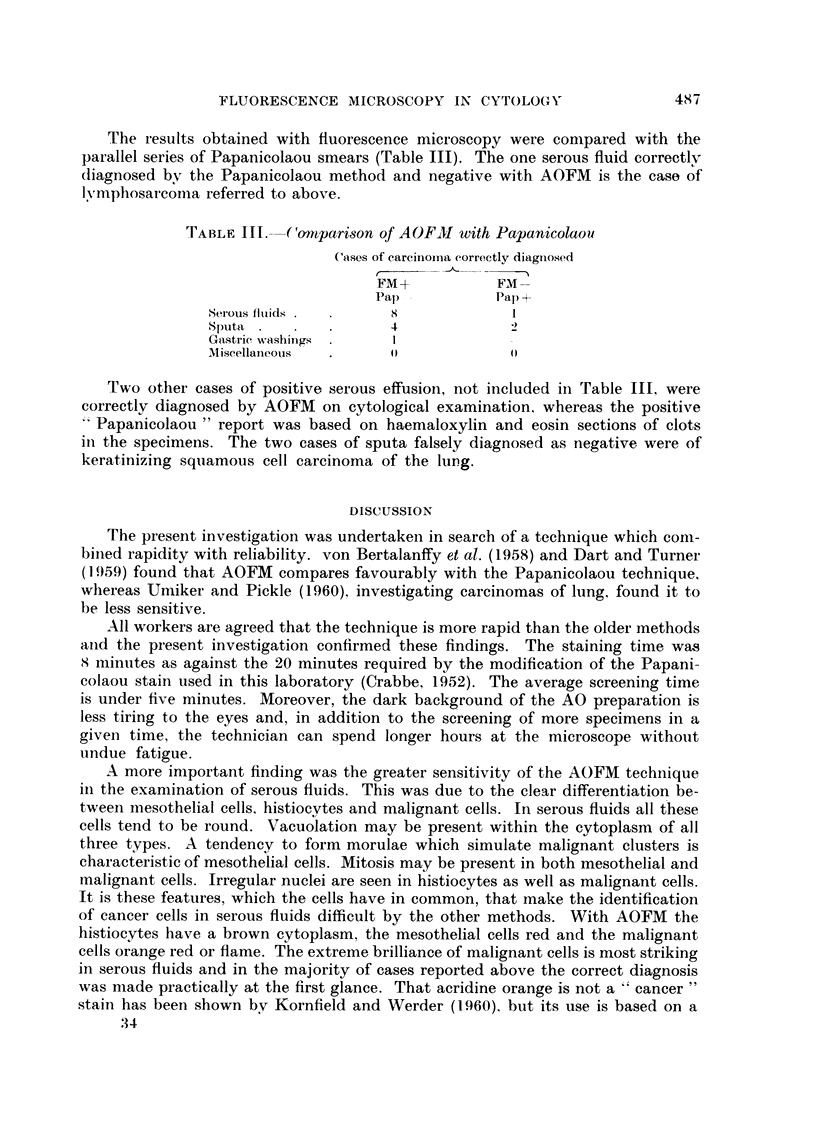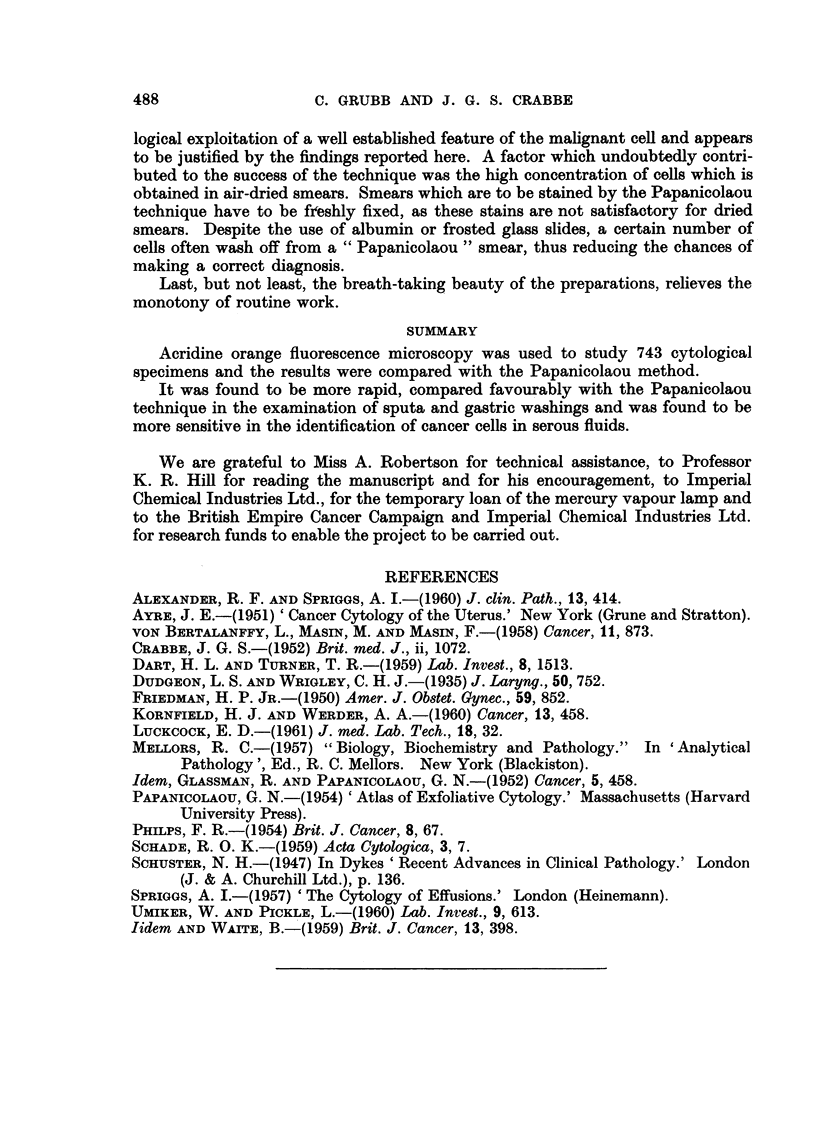# Fluorescence Microscopy in Exfoliative Cytology

**DOI:** 10.1038/bjc.1961.58

**Published:** 1961-09

**Authors:** C. Grubb, J. G. S. Crabbe

## Abstract

**Images:**


### FLUORESCENCE MICROSCOPY IN EXFOLIATIVE CYTOLOGY

C. GRUBB AND J. G. S. CRABBE

*From the Department of Pathology, Royal Free Hospital, Gray's Inn Road, London*

Received for publication May 5, 1961

EXFOLIATIVE cytology is a diagnostic procedure, which, having been generally accepted, is growing rapidly in importance as a means of early diagnosis of cancer. The Papanicolaou technique, developed originally to investigate gynaecological cancer, has been extensively applied to material from various sources and has collected a voluminous literature testifying to its usefulness, (Papanicolaou, 1954 ; Ayre, 1951 ; Schade, 1959). The diagnosis of the malignant cell however is, in the final analysis, subjective and it is experience and familiarity with a technique that is important, not the stain used. Philps (1954), using temporary wet films stained with methylene blue (Schuster, 1947) and permanent films stained with haemalum and eosin (Dudgeon and Wrigley, 1935), diagnosed 76·9 per cent carcinomas of lung by means of exfoliative cytology. Spriggs (1957) recommends May-Grundwald-Giemsa for the examination of serious fluids. Luckcock (1961), reporting from the Brompton Hospital, describes the use of Ehrlich's acid haematoxylin and eosin for the examination of tumour cells in blood, whereas, for the same purpose, Alexander and Spriggs (1960) find Romanowsky stains useful. The present study was undertaken to evaluate fluorescence microscopy for cytological diagnosis using acridine orange as described by von Bertalanffy, Masin and Masin (1958).

Fluorescence microscopy has been applied to cytology using berberine sulphate, acid fuchsin and the acridine group of stains (Friedman, 1950 ; Mellors, Glassman and Papanicolaou, 1952). Its use is based on the affinity of the basic fluorochrome dyes for the nucleic acids. Studies of the neoplastic cell have shown an increase in the total nucleic acids and ribonucleic acid (RNA). This increase is characteristic of cells showing a high protein synthesis and is seen in embryonic, regenerating, secretory and malignant cells. The RNA is found in the cytoplasm and the nucleoli of the affected cells. Increase in deoxyribonucleic acid (DNA), found in the chromatin of the nucleus, is seen in cells undergoing mitosis and polyploidy characteristic of advanced cancer. The fluorescent techniques described by Friedman and others have proved tedious and inadequate.

von Bertalanffy *et al.* (1958) have reviewed the literature on the role of nucleic acids in malignant cells, and the theoretical considerations for the use of basic fluorochrome stains and they have suggested the use of acridine orange in routine exfoliative cytology.

Acridine orange (AO) is a histochemical fluorochrome with a selective affinity for nucleic acids. At a concentration of 0·01 per cent and a pH of 6, recommended by von Bertalanffy, the DNA fluoresces yellow to whitish green, and the RNA red.

The differential fluorescence is due to varying degrees of polymerization of the nucleic acids, and makes possible the assessment of relative amounts of RNA and DNA in the cell. The increased RNA of the malignant cell is reflected in an increased brilliance, so that the differential fluorescence of the malignant and normal cells allows a comparison of the total concentration of nucleic acids in the various cells in the preparation. In addition, the morphological features of the cell are clearly visualised.

The advantages offered by this technique (hereafter named AOFM) are twofold. Firstly, the rapidity of processing and screening a specimen results in a saving of time, attractive to a rapidly growing and increasingly busy department. Secondly, it adds a certain amount of objectivity to the process of arriving at a diagnosis. These considerations led to an attempt to assess the value of this method in routine cytological diagnosis.

### MATERIAL AND METHODS

The method used is that described by von Bertalanffy *et al.* (1958). Dart and Turner (1959) and Umiker, Pickle and Waite (1959) have described various modifications which were found to be unnecessary.

The first two months were devoted to learning what amounted to a new language. A variety of specimens were stained and examined with AOFM. Different types of cells—normal, atypical and malignant were studied. They were subsequently decolourised in two changed of 50 per cent alcohol, restained with Papanicolaou stains and the results compared with those obtained with acridine orange. The specimens thus studied are not included in the present series. Thereafter, excepting gynaecological smears, all specimens coming to the laboratory, 743 in number, were examined by both techniques. Six smears were prepared from each specimen, three were screened by the Papanicolaou technique and three by fluorescence microscopy. The person screening the AO preparations did so without having looked at the corresponding Papanicolaou smears and the results were recorded independently as positive, suspicious or negative. After the first three months, one of the three acridine orange smears was air-dried before processing and then fixed and stained in the usual way.

### *Apparatus*

The Cook, Troughton, Simms mercury vapour lamp was used as a source of light. A blue exciter filter was fitted to the front of the lamp with a glass cell containing 5 per cent copper sulphate solution behind the filter. This could be replenished as and when necessary. Orange barrier filters were incorporated in both eye pieces.

A Cook, Troughton, Simms binocular microscope was used. Smears were screened using $\times 8$ oculars and $\times 5$ objective. In some cases of excessively cellular gastric washings and thick sputum smears, $\times 10$ objective had to be used for screening. Morphological detail was studied with a $\times 40$ objective.

The microphotographs were taken with a Leitz camera using super Anscochrome film. Trial and error showed that a 10–15 seconds exposure was adequate for a low power photograph and 20–30 seconds for a high power one.

*Cellular appearances*

The cellular appearances were similar to those described by von Bertalanffy *et al.* (1958) and Umiker and Pickle (1960) and are as follows (Table I). Mucus fluoresced green, bacteria orange and nucleoli when present were orange. The haemoglobin in red blood cells quenches fluorescence and they were not seen. The appearances of the cells in the air-dried preparations were identical with those that were freshly fixed. The description given above applies to a large majority of cases, but certain exceptions were encountered.

TABLE I.—*Appearances of Cells with AOFM*

| | Cytoplasm | Nucleus |
|---|---|---|
| | *Benign Cells* | |
| Leucocytes— | | |
| Polymorph neutrophils | Brown—barely visible | Green—occasionally whitish green |
| Lymphocytes | Red to orange | Yellow |
| Superficial squamous cells | Dull olive green | Green to white |
| Cells undergoing squamous metaplasia | Green — occasionally very dull reddish brown | Greenish white to yellow. |
| Histiocytes | Brown to brownish red—inclusions may be present | Green to yellow |
| Mesothelial cells | Red—generally brick—occasionally orange red | Dull yellow |
| Columnar cells | Reddish brown or brick red | Yellow-green. |
| | *Malignant Cells* | |
| | Cytoplasm | Nucleus |
| Keratinized squamous cells | Dull olive green | Bright yellow. |
| Poorly differentiated squamous cells | Reddish orange to flame | Bright yellow |
| Adeno-carcinoma | Orange to flame | Bright yellow or whitish green |
| Anaplastic carcinoma | Flame | Bright yellow or whitish green |

Necrotic malignant cells always had a dull brownish red cytoplasm, and the nucleus varied from bright yellow to dull yellowy green irrespective of the nature of the cell. The few cases, however, in which such cells were encountered came from cases of carcinomatosis and the morphological features made the diagnosis obvious (Fig. 1).

The cytoplasm of the keratinizing squamous cell was dull green or brown and liable to be overlooked. The nucleus, on the other hand, was extremely brilliant and arrested attention. The disparity between the fluorescence of the cytoplasm and nucleus of the keratinized cell had to be kept in mind if these were not to be missed (Fig. 2). A high power examination revealed the bizarre shapes commonly seen in well differentiated squamous cell carcinoma. In the midst of a large number of pus cells and histiocytes the bright nucleus would probably not be enough to focus attention on the cell.

In thick smears of sputum with large collections of histiocytes superimposed on each other the cells appeared excessively bright and the nuclei yellow. In such cases the diagnosis was based on an examination of the single cells at the edge of the group, with the appearances and staining features characteristic of histiocytes. In addition, the thick cluster generally showed carbon inclusions and was rejected for the purpose of diagnosis. Food particles fluoresced orange and yellow, but did not present a problem as they lack morphological characteristics of cells.

### RESULTS

The 743 specimens came from 395 cases which are summarised in Table II. The miscellaneous group includes CSF, breast secretions, bronchial washings and urines.

TABLE II.—*Results of AOFM and Papanicolaou Screening*

| | Serous fluids | | Sputa | | Gastric washings | | Miscellaneous | |
|---|---|---|---|---|---|---|---|---|
| | AOFM | Papanicolaou | AOFM | Papanicolaou | AOFM | Papanicolaou | AOFM | Papanicolaou |
| Total number of cases | 62 | 62 | 221 | 221 | 87 | 87 | 25 | 25 |
| True positives | 36 | 31 | 20 | 21 | 8 | 7 | 8 | 9 |
| True suspicious | 2 | — | 4 | 1 | 2 | 2 | 1 | 0 |
| False positive | 2 | 1 | 1 | 1 | 0 | 0 | 0 | 0 |
| False suspicious | 0 | 0 | 6 | 2 | 0 | 0 | 0 | 0 |
| False negative | 1 | 8 | 5 | 7 | 5 | 6 | 0 | 0 |
| Percentage cases of carcinoma correctly diagnosed | 97 | 79 | 82 | 76 | 67 | 60 | 100 | 100 |

The two cases (3 per cent) of serous fluids falsely diagnosed as positive were of congestive cardiac failure. The one false negative was found at autopsy to come from a case of lymphosarcoma. The cells in the effusion had in fact been diagnosed as being of the lymphocytic series, but it was not possible to say if they were malignant. Mellors (1957) has shown that DNA is not increased in human lymphoma and this could have been one reason why the cells with their narrow rim of cytoplasm were not recognized as being malignant, or at any rate, primitive.

Seven sputa were falsely diagnosed as being positive. Though this was only 4 per cent of the total number of cases examined, it represents 23 per cent of the total number of positives reported. These specimens were all from cases of bronchiectasis. It may be assumed that the exfoliated cells seen were hyperplastic and therefore fluoresced brightly. The morphological criteria of cells from cases of bronchiectasis are notoriously difficult to analyse and require greater experience than was available to us in the present survey.

### EXPLANATION OF PLATE

FIG. 1.—Ascitic fluid. Necrotic malignant cells showing dull brown red cytoplasm. Case of carcinomatosis. ×700.

FIG. 2.—Sputum. Malignant keratinizing squamous cells with dull green cytoplasm and bright yellow nuclei. Two undifferentiated cells have orange cytoplasm. ×700.

FIG. 3.—Sputum. Malignant cells showing differential staining and fluorescence. ×250.

FIG. 4.—Ascitic fluid. Showing differential staining and fluorescence of (*a*) malignant cells : bright orange cytoplasm with large yellow nucleus ; (*b*) mesothelial cells adjoining the malignant cells : dull brownigh red cytoplasm with yellow nucleus ; (*c*) two histiocytes in the centre of field : brown cytoplasm with green nuclei ; (*d*) polymorphs : lobulated green nucleus surrounded by very dull brown cytoplasm ; (*e*) lymphocytes : bright yellow dense nucleus with a rim of red cytoplasm. ×700.

FIG. 5.—Gastric lavage. Showing morphological features of malignant cells. Note the increase in nuclear size and the coarse clumping of chromatin and nucleoli. ×700.

FIG. 6.—Ascitic fluid. Showing a malignant cluster. Note the brilliance which facilitates screening. ×250.

1

2

3

4

5

6

The results obtained with fluorescence microscopy were compared with the parallel series of Papanicolaou smears (Table III). The one serous fluid correctly diagnosed by the Papanicolaou method and negative with AOFM is the case of lymphosarcoma referred to above.

TABLE III.—*Comparison of AOFM with Papanicolaou*

| | Cases of carcinoma correctly diagnosed | |
|---|---|---|
| | FM+ Pap− | FM− Pap+ |
| Serous fluids . . | 8 | 1 |
| Sputa . . . | 4 | 2 |
| Gastric washings . | 1 | – |
| Miscellaneous . | 0 | 0 |

Two other cases of positive serous effusion, not included in Table III, were correctly diagnosed by AOFM on cytological examination, whereas the positive " Papanicolaou " report was based on haemaloxylin and eosin sections of clots in the specimens. The two cases of sputa falsely diagnosed as negative were of keratinizing squamous cell carcinoma of the lung.

### DISCUSSION

The present investigation was undertaken in search of a technique which combined rapidity with reliability. von Bertalanffy *et al.* (1958) and Dart and Turner (1959) found that AOFM compares favourably with the Papanicolaou technique, whereas Umiker and Pickle (1960), investigating carcinomas of lung, found it to be less sensitive.

All workers are agreed that the technique is more rapid than the older methods and the present investigation confirmed these findings. The staining time was 8 minutes as against the 20 minutes required by the modification of the Papanicolaou stain used in this laboratory (Crabbe, 1952). The average screening time is under five minutes. Moreover, the dark background of the AO preparation is less tiring to the eyes and, in addition to the screening of more specimens in a given time, the technician can spend longer hours at the microscope without undue fatigue.

A more important finding was the greater sensitivity of the AOFM technique in the examination of serous fluids. This was due to the clear differentiation between mesothelial cells, histiocytes and malignant cells. In serous fluids all these cells tend to be round. Vacuolation may be present within the cytoplasm of all three types. A tendency to form morulae which simulate malignant clusters is characteristic of mesothelial cells. Mitosis may be present in both mesothelial and malignant cells. Irregular nuclei are seen in histiocytes as well as malignant cells. It is these features, which the cells have in common, that make the identification of cancer cells in serous fluids difficult by the other methods. With AOFM the histiocytes have a brown cytoplasm, the mesothelial cells red and the malignant cells orange red or flame. The extreme brilliance of malignant cells is most striking in serous fluids and in the majority of cases reported above the correct diagnosis was made practically at the first glance. That acridine orange is not a " cancer " stain has been shown by Kornfield and Werder (1960), but its use is based on a logical exploitation of a well established feature of the malignant cell and appears to be justified by the findings reported here. A factor which undoubtedly contributed to the success of the technique was the high concentration of cells which is obtained in air-dried smears. Smears which are to be stained by the Papanicolaou technique have to be freshly fixed, as these stains are not satisfactory for dried smears. Despite the use of albumin or frosted glass slides, a certain number of cells often wash off from a " Papanicolaou " smear, thus reducing the chances of making a correct diagnosis.

Last, but not least, the breath-taking beauty of the preparations, relieves the monotony of routine work.

### SUMMARY

Acridine orange fluorescence microscopy was used to study 743 cytological specimens and the results were compared with the Papanicolaou method.

It was found to be more rapid, compared favourably with the Papanicolaou technique in the examination of sputa and gastric washings and was found to be more sensitive in the identification of cancer cells in serous fluids.

We are grateful to Miss A. Robertson for technical assistance, to Professor K. R. Hill for reading the manuscript and for his encouragement, to Imperial Chemical Industries Ltd., for the temporary loan of the mercury vapour lamp and to the British Empire Cancer Campaign and Imperial Chemical Industries Ltd. for research funds to enable the project to be carried out.

### REFERENCES

ALEXANDER, R. F. AND SPRIGGS, A. I.—(1960) *J. clin. Path.*, **13**, 414.
AYRE, J. E.—(1951) ' Cancer Cytology of the Uterus.' New York (Grune and Stratton).
VON BERTALANFFY, L., MASIN, M. AND MASIN, F.—(1958) *Cancer*, **11**, 873.
CRABBE, J. G. S.—(1952) *Brit. med. J.*, ii, 1072.
DART, H. L. AND TURNER, T. R.—(1959) *Lab. Invest.*, **8**, 1513.
DUDGEON, L. S. AND WRIGLEY, C. H. J.—(1935) *J. Laryng.*, **50**, 752.
FRIEDMAN, H. P. JR.—(1950) *Amer. J. Obstet. Gynec.*, **59**, 852.
KORNFIELD, H. J. AND WERDER, A. A.—(1960) *Cancer*, **13**, 458.
LUCKCOCK, E. D.—(1961) *J. med. Lab. Tech.*, **18**, 32.
MELLORS, R. C.—(1957) "Biology, Biochemistry and Pathology." In ' Analytical Pathology ', Ed., R. C. Mellors. New York (Blackiston).
*Idem*, GLASSMAN, R. AND PAPANICOLAOU, G. N.—(1952) *Cancer*, **5**, 458.
PAPANICOLAOU, G. N.—(1954) ' Atlas of Exfoliative Cytology.' Massachusetts (Harvard University Press).
PHILPS, F. R.—(1954) *Brit. J. Cancer*, **8**, 67.
SCHADE, R. O. K.—(1959) *Acta Cytologica*, **3**, 7.
SCHUSTER, N. H.—(1947) In Dykes ' Recent Advances in Clinical Pathology.' London (J. & A. Churchill Ltd.), p. 136.
SPRIGGS, A. I.—(1957) ' The Cytology of Effusions.' London (Heinemann).
UMIKER, W. AND PICKLE, L.—(1960) *Lab. Invest.*, **9**, 613.
*Iidem* AND WAITE, B.—(1959) *Brit. J. Cancer*, **13**, 398.